# From Embryos to Adults: A DOHaD Perspective on In Vitro Fertilization and Other Assisted Reproductive Technologies

**DOI:** 10.3390/healthcare4030051

**Published:** 2016-08-09

**Authors:** Sky Feuer, Paolo Rinaudo

**Affiliations:** Department of Obstetrics, Gynecology and Reproductive Sciences, University of California at San Francisco, San Francisco, CA 94143, USA; sky.feuer@ucsf.edu

**Keywords:** DOHaD, assisted reproductive technologies, in vitro fertilization, reprogramming

## Abstract

Human in vitro fertilization (IVF) as a treatment for infertility is regarded as one of the most outstanding accomplishments of the 20th century, and its use has grown dramatically since the late 1970s. Although IVF is considered safe and the majority of children appear healthy, reproductive technologies have been viewed with some skepticism since the in vitro environment deviates substantially from that in vivo. This is increasingly significant because the Developmental Origins of Health and Disease (DOHaD) hypothesis has illuminated the sensitivity of an organism to its environment at critical stages during development, including how suboptimal exposures restricted specifically to gamete maturation or the preimplantation period can affect postnatal growth, glucose metabolism, fat deposition, and vascular function. Today, some of the physiological metabolic phenotypes present in animal models of IVF have begun to emerge in human IVF children, but it remains unclear whether or not in vitro embryo manipulation will have lasting health consequences in the offspring. Our expanding knowledge of the DOHaD field is fueling a paradigm shift in how disease susceptibility is viewed across the life course, with particular emphasis on the importance of collecting detailed exposure information, identifying biomarkers of health, and performing longitudinal studies for any medical treatment occurring during a developmentally vulnerable period. As IVF use continues to rise, it will be highly valuable to incorporate DOHaD concepts into the clinical arena and future approaches to public health policy.

## 1. Introduction: The Triumph of Reproductive Technologies and in Vitro Fertilization

The evolution of assisted reproductive technologies (ART) over the past decades has incited a worldwide reproductive revolution, providing tremendous hope and joy not only to the ~15% of couples experiencing fertility problems at least once during their reproductive lifetimes, but also to oncology patients, same sex partners, individuals without a partner, couples facing heritable genetic diseases, and those wishing to accelerate or delay family planning. Approximately 1.5 million ART cycles are performed annually worldwide, resulting in an estimated 350,000 babies born [1]. At its outset, in vitro fertilization (IVF, accounting for >99% of all ART procedures) was regarded as a maverick technology, confronted with skepticism, hesitancy, and grave concern over unknown health consequences in any potential offspring [2]. However, the initial successes and resulting healthy children in the late 1970s quickly replaced these fears with enthusiasm, and IVF is now recognized as a hallmark medical advancement that has resulted in the birth of over five million babies as of 2012 [3]. As the utilization of reproductive technologies expands, newer procedures have involved increased embryo manipulation in vitro, such as intracytoplasmic sperm injection (ICSI), oocyte or embryo cryopreservation and thawing, and preimplantation genetic testing.

## 2. Preimplantation Development and the DOHaD Hypothesis

The Developmental Origins of Health and Disease (DOHaD) field has provided appreciable evidence that exposure to stressful environments in early development can increase an organism’s susceptibility to chronic, non-communicable diseases later in life. The hypothesis postulates that during critical periods in development—including periconception, pregnancy, and early postnatal life—organisms exhibit an enhanced plasticity that enables them to fine-tune patterns of gene expression in accordance with environmental cues. This so-called “reprogramming” thereby engenders an ability to adapt to novel conditions and diverse surroundings [4]. However, while re-shaping developmental trajectories can confer immediate survival advantages, these adaptive changes can conflict with postnatal environments and impair adult heath [5,6,7,8]. Chronic diseases are predicted to occur if there exists a mismatch between the environment experienced during development (for example, low energy availability) and the adult environment (such as food overabundance, common in many developed countries). Indeed, ischemic heart disease, stroke, diabetes, obesity, and other latent cardiometabolic pathologies have been widely observed following environmental stresses experienced in early development.

To date, several laboratories have used experimental animal models to show that manipulating the environment during the periconceptual period—both in vivo and in vitro—can have significant long-term consequences on growth and metabolism in the resulting offspring. A groundbreaking study demonstrated that a maternal low protein diet administered exclusively during preimplantation development resulted in offspring manifesting altered postnatal growth and hypertension [9]. This is not surprising, as preimplantation development is a period of elegantly orchestrated molecular milestones, including cell proliferation, differentiation, and extensive epigenetic reorganization (reviewed in [10]). The embryo’s evolving developmental and metabolic requirements occur coincidently with stage-specific fluctuations in nutrient availability, oxygen tension, and pH. As a result, although embryo metabolic plasticity is adept at compensating for nutrient flux, perturbation to the preimplantation environment can have widespread effects on cell number, growth velocity, metabolic homeostasis, and gene expression profiles (reviewed in [11,12,13]). Depending upon the timing, type, and degree of environmental perturbation, the developing embryo can be differentially affected and undergo adaptive changes, leading to concomitant metabolic derangements in the cells that subsequently form tissues and organ systems later in development.

Fertilization and embryo culture in vitro present a nutritional, biochemical, and hormonal environment that deviates significantly from natural conception. Not only are the dynamic conditions existent within the genital tract lost with IVF, but the procedure introduces several novel variables—high oxygen levels, mechanical stress, unique culture medium compositions, fluctuations in temperature and pH—such that the preimplantation embryo may have limited mechanisms for maintaining homeostasis when challenged with these stimuli [14]. In accordance with the DOHaD hypothesis [15], embryo defense strategies against in vitro stress exposures could affect the programming of cell states and signaling pathways, leading to aberrant responses to normal physiological signals, exhaustion of cell machinery, and long-term metabolic phenotypes. However, given that the oldest IVF-conceived person is only 38 years old, the potential effects of ART procedures on health and disease susceptibility in later adulthood are yet to be observed.

## 3. Outcomes of IVF

### 3.1. Animal Evidence

Animal models are advantageous not only for developing and improving ART procedures, but are especially valuable for investigating their impact on acute and, in particular, long-term health. Importantly, the use of animal models removes fertility as a confounding factor, permitting in-depth analyses of the potential effects of ART techniques without the variability related to infertility. Infertility in humans can additionally be a secondary manifestation of another medical condition (for example endometriosis)—which itself can have genetic or epigenetic etiology—further complicating the interpretation of data. A caveat is that the requirements for fertilization and preimplantation development can vary across different mammalian species, providing an incomplete or inaccurate extrapolation of the demands of the human embryo [16].

Mouse models investigating the long-term effects of IVF have reported a range of outcomes. Overall, changes in postnatal phenotype are increasingly severe as the in vitro conditions deviate more extensively from the natural reproductive tract [17,18,19]. For example IVF performed under suboptimal conditions (Whitten’s medium and 20% oxygen saturation, similar to the earliest years of human IVF) alters postnatal growth and impairs glucose tolerance in both male and female mice. In comparison, IVF optimized using current clinical conditions (K simplex optimized medium and 5% oxygen concentration) precipitates a latent and female-specific effect: growth kinetics and glucose tolerance are indistinguishable between IVF and control mice until approximately 17 weeks of age, at which point IVF females become heavier, display increased adiposity, and exhibit signs of β-cell dysfunction and hyperinsulinemia [20]. The same IVF model performed in a different mouse strain found a male-specific effect of suboptimal culture conditions on growth, glucose tolerance, and insulin resistance, whereas optimized culture conditions normalize offspring growth and metabolic phenotypes [21]. Other animal evidence corroborates that environmental stress in vitro can impair adult glucose tolerance and β-cell function, and in some cases increase adiposity or impair vascular function and longevity [22]. Although detrimental metabolic phenotypes have been observed in IVF mice compared to naturally-conceived controls, these outcomes can be moderated by sex, genetics, tissue type, the specific conditions of fertilization or embryo culture, as well as nutrition in pregnancy. These studies indicate that preimplantation plasticity is complex, and that effects of IVF procedures in humans might only be revealed in longitudinal population studies.

Reprogramming and developmental plasticity are believed to be mediated by epigenetic changes, and there is abundant evidence that in vitro manipulation affects the epigenetic landscape in embryos [23]. Given that chromatin is extensively reorganized during preimplantation development [24], it is possible that IVF-induced changes in transcriptional and epigenetic regulation are responsible for propagating adult metabolic phenotypes. Most importantly, while short-term effects have been extensively described [25,26], the clinical significance of any potential long-term effects of IVF on resulting offspring remains to be determined in humans.

### 3.2. Epidemiological Evidence

Several reviews describing epidemiological evidence of DOHaD principles, as well as obstetric and pediatric IVF outcomes are available [19,27,28,29]. Therefore, only data relevant to both ART and the DOHaD conceptual framework will be discussed here. First, multiple gestations, low birth weight, and preterm birth are well-described perinatal complications of ART [30,31]. Although risk for low birth weight and preterm birth is highest in multiples, their increased frequency in singleton IVF pregnancies indicate that some abnormalities in growth and placental development are present. Low birth weight is an established (but not robust [32]) marker of intrauterine stress and has been associated with coronary heart disease, hypertension, and hyperlipidemia [33], as well as increased blood pressure, fasting insulin concentrations, insulin resistance, and incidence of type 2 diabetes [34]. Additionally, ART procedures can predispose to fetal growth restriction in early to mid-pregnancy, followed by significant increases in placental size and rapid fetal growth towards the end of gestation (reviewed in [35]). This accelerated ‘catch-up’ growth can predispose to cardiometabolic complications in adulthood [36], and compound the latent cardiovascular risks of low birth weight [37].

The long-term health of IVF children is arguably the most pressing question of the field today, although the mechanisms underlying the relationship between preimplantation embryo manipulation and adult-onset pathologies are elusive. IVF-conceived adolescents present modest yet significant differences in growth kinetics [38], fat deposition [39], blood pressure, and glucose levels [40] compared to children conceived spontaneously by subfertile parents (defined as spontaneous conception after ≥1 year of infertility). Subfertility constitutes a valuable control group, and is the most ethical way to account for the confounding effects of different fertility problems. Another study observed ART-induced differences in systemic circulation using surrogate markers of pulmonary vascular function following day 2 transfer of IVF embryos cultured in media lacking amino acids, indicating that the 2–4 celled embryo is also vulnerable to environmental stress [41]. This study additionally highlights how early symptoms of systemic and pulmonary vascular impairments may follow culture in vitro. Of note, these changes are subtle and within the normal range, but may indicate a predisposition to cardiometabolic irregularities in adulthood.

## 4. A Brief Overview of Current ART Regulations

IVF and other assisted reproductive procedures are available all over the world, and most countries have adopted a mix of dedicated legislation and/or guidelines concerning the utilization of ART treatments. These regulations exhibit considerable regional differences that reflect the myriad of cultural, religious, and moral preferences surrounding ART practices. Indeed, the ethical complexities inherent in IVF converge upon the fundamental subjects of life, family, and society, which are enmeshed within different societal value systems [42,43]. As such, ART regulations fluctuate internationally between stringent management of specific procedures to lack of any political oversight, reflecting the diverse boundaries between government and faith or tradition in different cultures. Some countries have created specific regulatory entities that oversee all matters related to reproductive technologies, for example the United Kingdom (UK); others have scattered mechanisms of regulation in which assorted aspects of ART (genetic testing, reporting of success rates, use of medical devices, etc.) are each controlled by different agencies, as occurs in the United States (USA) [44,45].

Worldwide, there are several centralized reporting registries that monitor ART demographics and publish ART outcomes, which are continuously redesigned to reflect advances in IVF technology. Because reporting is not compulsory, participation in these registries (by clinic or country) is variable. Unfortunately, this is a tremendous barrier to capturing statistics describing the success rates and relevance of novel, innovative technologies in the ART field. For example, the most recent report from the European IVF-Monitoring Consortium contains data from 33 of 51 European countries and approximately 81% of clinics [46]. In the USA, the Fertility Clinic Success Rate and Certification Act of 1992 mandates that all clinics provide a variety of data annually to the Center for Disease Control and Prevention (CDC, [47]). However, there are no penalties for clinics failing to comply with the law other than the names of noncompliant clinics being listed in the appendix of the CDC’s annual report on ART success rates.

## 5. How DOHaD Thinking Can Influence ART Policy

ART procedures were developed specifically as a medical intervention for infertility, therefore the ultimate goals are achievement of a healthy pregnancy and the health of resulting children. Under the umbrella of the DOHaD theory, there are new and highly relevant considerations for the safety and efficacy of ART procedures that should be incorporated into ART regulatory schemes, which are discussed below.

### 5.1. Longitudinal Studies Are Essential

The DOHaD conceptual framework has demonstrated that environmental stress during early development can negatively affect an individual’s health in adulthood, which suggests that a long-term mindset must be applied to evaluating the outcome of any medical intervention, particularly if conducted during a period such as periconception. Therefore, in addition to the primary outcome of ART procedures (i.e., the delivery at term of a live, healthy singleton), the postnatal effects should be studied beyond the neonatal period. Unfortunately, longitudinal studies are costly, time-consuming, and are complicated by the logistically taxing processes of long-term (>years) recruitment and retention of large treatment populations. Most funding mechanisms are not supportive of these studies, particularly if no phenotypes are immediately present.

This issue may be partially circumvented in two ways. First, the use of animal models should be expanded as a valuable and necessary tool for evaluating long-term outcomes. Second, early biomarkers predictive of future health should be identified and carefully monitored in both animal models and human population studies. For example, incidence of preterm birth, low birth weight, and accelerated catch up growth are correlated with poorer cardiometabolic health in adulthood, including increased risk of developing hypertension, diabetes, obesity, and other symptoms of metabolic syndrome. However, birth weight—a supposed marker of fetal growth—is not always a reliable indicator of fetal health or stress in IVF pregnancies [32,35,48,49]. One promising molecular tool is the measurement of telomerase expression and telomere length in ART children, as shorter telomeres are a predictive marker of cardiovascular dysfunction, metabolic syndrome, and other age- and DOHaD-related pathologies [50,51]. As researchers continue identifying additional diagnostic biomarkers of future chronic disease, animal studies will be vital for comparing new markers with subsequent health outcomes in adult ART offspring.

### 5.2. Health Policies Should Focus on Addressing Chronic, Adult-Onset Metabolic Diseases

Exposure to harmful environments during vulnerable periods in development can significantly impact susceptibility to chronic metabolic diseases such as diabetes, hypertension, and obesity in adulthood. Cardiovascular diseases are the primary cause of death worldwide, and chronic disease-related deaths continue to rise [52]. This suggests that regulations involving medical interventions that occur within sensitive developmental windows—such as IVF—should incorporate the latent disease risk potential into any evaluation of safety and efficacy. For example, the USA’s Food and Drug Administration (FDA) has authority to address key disease transmission concerns raised by medical treatments involving human cells, tissues, and cellular- and tissue- based products (HCT/Ps), which include reproductive tissues, gametes and embryos [53,54]. HCT/Ps are organized under two different regulatory schemes, determined broadly by their level of manipulation and intended use [55]. Conventional ART procedures (including ICSI) are classified under the FDA’s definition of minimal manipulation, which is assumed not to alter the ‘relevant biological characteristics of cells or tissues’. As such, their regulation dictates that (1) they are governed by directives aimed at the prevention of communicable diseases; and (2) products used for the preparation, maintenance, transfer or storage of human gametes or embryos (i.e., gamete and embryo culture media and organic supplements) are not subject to premarket approval. The second regulatory scheme covers procedures considered to involve ‘more than minimal’ manipulation: only ART techniques involving the transfer of genetic material—for example oocyte nuclei or mitochondrial genetic material in ooplasm—require pre- and post-market review to demonstrate their safety and efficacy [56].

Previous DOHaD studies have demonstrated that omitting a focus on long-term chronic and non-communicable disease risk compromises patient safety. Medical interventions administered within a vulnerable period of development warrant special consideration due to the potential long-term disease risk, and the relevant governing policies should be expanded to include this theoretical focus. Therefore, pre-market approval and post-market follow up become increasingly valuable for any new technology, including for improvement and supplementation of reagents used in embryo culture.

### 5.3. Reported IVF Outcomes Must Capture the Details of the Preimplantation Environment

The accumulating literature on animal models (and preliminary human data) indicates that embryo, neonatal and long-term outcomes are modifiable by specific in vitro embryo culture conditions. Animal data demonstrate that even modest variations in the individual components of embryo manipulation can have pronounced effects on blastocyst gene expression, imprinted gene methylation and expression [57,58], birth weight, postnatal growth, and adult glucose metabolism [17,20,21,59,60,61]. It would therefore be highly valuable to monitor obstetric and prenatal outcomes stratified by the use of ICSI versus conventional IVF, preimplantation genetic diagnosis (PGD) versus preimplantation genetic screening (PGS), culture medium composition and oxygen concentration, day of transfer (day 2–5), sperm origin (testicular, epididymal, or ejaculated), ovarian stimulation protocol, as well as the use of cryopreservation or assisted hatching. In accordance with the DOHaD hypothesis, each of these parameters could have a distinct and potentially compounding effect on offspring growth and metabolism. Accordingly, it is crucial to further redesign the centralized reporting registries in order to capture as many elements of ART procedures as possible, because detailed information pertaining to the embryo fertilization and culture environments is currently either omitted or pooled in ART cycle results. ART statistics are difficult to comprehensively assess on a broad scale, but generalizing or pooling data can introduce noise and potentially obscure reported outcomes. Moreover, in spite of recommendations for best clinical practice, the embryo culture environment is not standardized [62].

### 5.4. Technological Innovations and New Health Data Will Require Flexibility of the Overseeing Agencies

The DOHaD paradigm is changing what scientists believe is adequate evidence to support the effectiveness of a new intervention. Health promotion and prevention programs will benefit greatly from incorporating this mindset into clinical care. Two relevant examples are the recent development of mitochondrial replacement techniques and oocyte cryopreservation.

#### 5.4.1. Mitochondrial Replacement Techniques

Mitochondrial replacement techniques (MRTs) were developed for patients carrying mitochondrial diseases, which affect approximately 1 in 4000 individuals and vary significantly in their presentation and severity. These techniques—which include maternal-spindle transfer, pronuclear transfer, and polar body transfer—involve substituting the maternal mitochondria in oocytes with mitochondria from a healthy donor to prevent the transmission of mitochondrial DNA (mtDNA) diseases from mother to child. As such, MRTs require significant manipulation of gametes in vitro and would result in offspring with genetic material from three separate lineages (i.e., sperm and oocyte DNA, as well as mitochondrial DNA from the healthy donor).

MRTs have recently reached new relevance following the proposal to transfer younger mitochondria to aging oocytes using organelles extracted from the patient’s own ovarian tissue [63]. The proponents of this approach argue that because this strategy improves oocyte quality, it should be made widely available (as opposed to its current use in rare patients with mitochondrial disease). Of note, the mitochondrial transfer was first attempted in the 1990s [64,65] but soon abandoned out of concern over risks for heteroplasmy, chromosome abnormalities, and pervasive birth or developmental defects [66,67]. After many technological advances, today MRTs are approved for ART patients with mitochondrial disease in the UK, and are still under consideration in other countries [44].

Clinical feasibility of MRT and maternal-spindle transfer (ST) was pioneered in rhesus macaque, a non-human primate model [68,69]. A three-year follow up of monkey ST offspring showed no mitochondrial heteroplasmy or dysfunction, but a close examination of the their growth kinetics revealed that ST juveniles exhibited exaggerated catch up growth: they were born weighing nearly 2 standard deviations below controls and within four months had grown to nearly 2 standard deviations above control weights [69]. This accelerated weight gain is a significant marker of adult metabolic syndrome (reviewed in [70]), but was not discussed in the article. This underscores the importance of analyzing postnatal outcomes with a DOHaD lens, and suggests that more studies are necessary to address whether mitochondrial spindle-transfer (or the IVF procedure itself) will affect adult cardiometabolic health.

#### 5.4.2. Oocyte Cryopreservation

Oocyte cryopreservation has gained increasing popularity since 2012, after the American Society for Reproductive Medicine (ASRM) lifted the ‘experimental’ label for the procedure [71]. It is difficult to obtain robust numbers on its prevalence worldwide: between 2009 and 2013, the number of women in the USA choosing to freeze their eggs grew seven-fold [72], and fertility marketer Eggbanxx estimates that 76,000 women will have frozen their eggs by 2018 [73]. However, both the ASRM and the American College of Obstetricians and Gynecologists (ACOG) caution against the marketing of egg freezing for elective purposes (i.e., oocyte cryopreservation to preserve fertility potential, without a medical indication), warning of insufficient data addressing outcomes related to healthy women wishing to circumvent reproductive aging, and that voluntary oocyte cryopreservation might offer false hope and encourage women to delay childbearing [71,74]. Indeed, infertile couples face a straightforward risk/benefit ratio when choosing ART, in that any risks associated with the use of IVF to treat infertility are balanced by the benefit of conceiving a child. Opening this technology to the fertile population alters this ratio and thus has considerable implications for future ART patients.

Nevertheless, oocyte cryopreservation has become a rapidly growing market for young, fertile women. Egg banking companies sponsor ‘egg freezing’ parties with catchy names such as ‘Let’s Chill’ or ‘Wine and Freeze’ to both incentivize and educate about fertility preservation [75]. Although touted as an insurance policy to revitalize waning fertility, many agree that the industry focuses on women’s insecurities by marketing the promise that freezing eggs is the modern woman’s responsibility [76]. Further, while several companies previously covered oocyte cryopreservation costs for employees preparing to undergo chemotherapy, tech giants Facebook and Apple recently announced social egg freezing employee benefits of up to $20,000 [77]. In January 2016, the United States Department of Defense announced egg freezing coverage for its active duty service women, as part of their Force of the Future Reform [78]. Several investigators hold that company-sponsored egg freezing limits women’s reproductive autonomy [79].

### 5.5. The Importance of Abiding by Recommendations for Best Practice

Assisted reproductive technologies pose a unique challenge to the policy arena because the field continues to rapidly improve and grow. New treatments and more sophisticated approaches are quickly adapted into clinical practice. Yet in many countries, clinical services are largely subject to professional self-regulation. As a result several non-governmental organizations—for example, the International Federation of Fertility Societies (IFFS), American Society for Reproductive Medicine (ASRM), and the European Society of Human Reproduction and Embryology (ESHRE)—have released a multitude of committee-based recommendations for best ART practices. These are particularly valuable for the adoption of novel technologies not yet regulated. Separately, these guidelines are beneficial for larger countries such as Australia or the USA, where efforts to avoid conflict between state autonomy and federal oversight has resulted in a highly fragmented, patchwork-like system of ART legislation [80].

However, adherence to industry guidelines is strictly voluntary, and current practices can diverge from professional recommendations. In addition to the recommendations against elective oocyte cryopreservation [71,74], a good example concerns the use of intracytoplasmic sperm injection (ICSI). ICSI was initially developed to treat severe male infertility and uses a micromanipulation device to inject a single sperm into an oocyte. ICSI therefore involves significant mechanical manipulation of gametes and also bypasses several biological barriers that naturally select for optimal fertilization. There is overwhelming evidence that IVF birth outcomes using ICSI versus conventional insemination are similar [81], with few studies suggesting an increased risk of birth defects [82] or altered metabolic parameters in the offspring [83]. However, ICSI is associated with blastocyst growth retardation and considerable gene misexpression compared to conventional IVF [61], and animal models suggest that greater blastocyst gene misexpression—secondary to increasingly stressful embryo culture conditions—is associated with more severe metabolic phenotypes in adulthood [17,20,84]. It remains unclear whether this is relevant to human IVF, as the first successful birth by ICSI occurred in 1992.

ICSI also increases both the complexity and cost of IVF: it requires additional laboratory experience, technical expertise, resources, effort, and time. Today, ICSI is used in 67% of all ART cycles worldwide [1,72], despite a lack of evidence that ICSI provides any clinical advantage (including increased fertilization rate, clinical pregnancy rate, or live birth rate) in patients with non-male factor infertility or a history of prior fertilization failure [85]. There is no data justifying the routine use of ICSI, including for patients with unexplained infertility or low oocyte yield. In fact, the American Society for Reproductive Medicine purposely does not support the indiscriminate use of ICSI in all IVF cycles [85].

## 6. Conclusions: Moving Innovation to Practice

Many infertile couples who pursue ART are unaware that the long-term outcomes of IVF are unknown, and that the preimplantation environment is an exquisitely vulnerable period with the potential to influence their children’s future health. It is therefore paramount that health providers be responsible for educating and counseling patients on this topic. However, while many studies specify the importance of the periconceptual environment, this information is often not available to clinicians in a straightforward way. Very appropriate to this conversation is the following statement by T. Woodruff about the challenges of disseminating information about environmental health sciences:

“One factor that makes it difficult for clinicians, patients, and policy makers to make use of [the] science is that it is not systematically and transparently evaluated and synthesized in a timely manner. The scientific evidence is voluminous, of variable quality, and largely unfamiliar to health professionals caring for people of childbearing age. And there is no trusted ready reference or compendium that provides health professionals and patients with timely, evidence-based advice about exposure [to environmental contaminants]” [86]. 

Here, assisted reproductive technologies are no different. Innovation is a fundamental component of improving health care [87], and as such it is essential that advances in the field are properly communicated to all parties. This can manifest as continuing education credits for the professional development of health care providers, and additionally with thorough and detailed reporting of statistics that differentiate between specific ART conditions and link to postnatal long-term health outcomes.

DOHaD paradigms are reshaping how health policies are approached by researchers and ART treatment providers. The rapid evolution of IVF technologies has long excited media attention, but it is exceedingly important to remain mindful that critically vulnerable stages of development such as the preimplantation period can alter growth and metabolic trajectories across the life course. These effects may not manifest for several years or even decades, further emphasizing the importance of long-term follow up of any novel (and DOHaD-relevant) technologies before their clinical implementation. The simple, powerful, and dangerous truth is that the long-term effects of in vitro fertilization, ICSI, and embryo culture on resulting offspring are unknown—with evidence both supporting and refuting detrimental outcomes. It is possible that IVF offspring will remain perfectly healthy into adulthood, or conversely they might be predisposed to chronic diseases. Further, if IVF-conceived offspring are predisposed to chronic diseases, it will be challenging to impute it to the use of ART because several lifestyle variables such as diet and stress can impact the molecular mechanisms that underlie metabolic diseases.

The precautionary principle that an absence of harm is not evidence of no harm is especially relevant within the DOHaD framework, where phenotypes often manifest latently. As a result, even the most complex animal IVF data should not be dismissed. Rather, multifaceted data should incentivize that more evidence—both animal and human—be gathered. Arguably the amount of evidence required for precautionary action is both an ethical choice and a scientific issue, and can only be decided by continuous collaboration between scientists, clinicians, community members and political groups. Therefore, it is our charge as a research community to address these critical questions and early warnings within the ART field. With the growing opportunities available through assisted reproduction, including oocyte cryopreservation for young women or mitochondrial replacement therapies, it is imperative to incorporate DOHaD concepts into the clinical arena and future approaches to public health policy.

## Abbreviations

The following abbreviations are used in this manuscript:

DOHaD	Developmental origins of health and disease
ART	Assisted reproductive technologies
IVF	In vitro fertilization
ICSI	Intracytoplasmic sperm injection
PGD	Preimplantation genetic diagnosis
PGS	Preimplantation genetic screening
MRT	Mitochondrial replacement therapy
FDA	Food and Drug Administration
CDC	Center for Disease Control
HCT/Ps	Human cells, tissues, and cellular and tissue-based products
ASRM	American Society for Reproductive Medicine
ACOG	American College of Obstetricians and Gynecologists
ESHRE	European Society of Human Reproduction and Embryology
IFFS	International Federation of Fertility Societies
